# Urine metabolomics analysis of sleep quality in deep-underground miners: A pilot study

**DOI:** 10.3389/fpubh.2022.969113

**Published:** 2022-08-19

**Authors:** Qiao Wen, Jing Zhou, Xiaoru Sun, Tengfei Ma, Yilin Liu, Yike Xie, Ling Wang, Juan Cheng, Jirui Wen, Jiang Wu, Jian Zou, Shixi Liu, Jifeng Liu

**Affiliations:** ^1^Department of Otolaryngology Head and Neck Surgery, West China Hospital, Sichuan University, Chengdu, China; ^2^Deep Underground Space Medical Center, West China Hospital, Sichuan University, Chengdu, China; ^3^Department of Ophthalmology, West China Hospital, Sichuan University, Chengdu, China

**Keywords:** sleep quality, deep-underground medicine, urine, metabolomics, biomarker

## Abstract

**Background:**

In previous questionnaire surveys of miners, sleep disorders were found among underground workers. The influence of the special deep-underground environment and its potential mechanism are still unclear. Therefore, this study intends to utilize LC-MS metabolomics to study the potential differences between different environments and different sleep qualities.

**Methods:**

Twenty-seven miners working at 645–1,500 m deep wells were investigated in this study, and 12 local ground volunteers were recruited as the control group. The Pittsburgh Sleep Quality Index (PSQI) was used to examine and evaluate the sleep status of the subjects in the past month, and valuable basic information about the participants was collected. PSQI scores were obtained according to specific calculation rules, and the corresponding sleep grouping and subsequent analysis were carried out. Through liquid chromatography-mass spectrometry (LC-MS) non-targeted metabolomics analysis, differences in metabolism were found by bioinformatics analysis in different environments.

**Results:**

Between the deep-underground and ground (DUvsG) group, 316 differential metabolites were identified and 125 differential metabolites were identified in the good sleep quality vs. poor sleep quality (GSQvsPSQ) group. The metabolic pathways of Phenylalanine, tyrosine and tryptophan biosynthesis (*p* = 0.0102) and D-Glutamine and D-glutamate metabolism (*p* = 0.0241) were significantly enriched in DUvsG. For GSQvsPSQ group, Butanoate metabolism was statistically significant (*p* = 0.0276). L-Phenylalanine, L-Tyrosine and L-Glutamine were highly expressed in the deep-underground group. Acetoacetic acid was poorly expressed, and 2-hydroxyglutaric acid was highly expressed in good sleep quality.

**Conclusions:**

The influence of the underground environment on the human body is more likely to induce specific amino acid metabolism processes, and regulate the sleep-wake state by promoting the production of excitatory neurotransmitters. The difference in sleep quality may be related to the enhancement of glycolytic metabolism, the increase in excitatory neurotransmitters and the activation of proinflammation. L-phenylalanine, L-tyrosine and L-glutamine, Acetoacetic acid and 2-hydroxyglutaric acid may be potential biomarkers correspondingly.

## Introduction

The consumption of the earth's shallow resources has been accelerated due to the rapid population growth, which brought unprecedented challenges to the sustainable development (1, 2). Researchers worldwide are progressed to make efforts in directions, such as space, underground, deep sea, or even the pole, where researches and explorations are conducted for survival and constant development (1, 3, 4). However, little is known with regard to the underground biology, especially in a deep environment over 1,000 m.

Thus far, deep-underground medicine (DUGM) was put forward by an expert team led by Academician Xie Heping after full discussion and demonstration from 2015 to 2017 (2). Deep-underground medicine refers to study of the physiological, pathological and psychological effects of living organisms in deep underground, especially those with behavioral-cognitive ability, and their response mechanism under different underground environmental conditions. Furthermore, DUGM also contains the coping strategies of harmful factors, as well as the safe and efficient use of underground beneficial factors to serve human social activities. The DUGM integrated the research data collected from the open deep-underground space, tunnels and the simulation capsule (1, 2).

Previous studies have confirmed the changing patterns of various living organisms in this special deep-underground environments, including protozoa (*Paramecium tetraurelia* and *Synechococcus lividus*) (1, 5), *Saccharomyces cerevisiae* (6), mammalian cells (V79, FD-LSC-1) (4, 7), and *Drosophila melanogaster* (8, 9). Planel, Kawanishi, and Castillo described that cell proliferation was slowed down (5, 10, 11). Morciano found that Drosophila melanogaster's life span was prolonged and its reproductive capacity was decreased (8). More recently, Antonelli and Carbone pointed out that the cell's response ability to DNA damage was reduced (12–14). However, follow-up studies reported a dissimilar phenomenon under below background radiation (BBR). Wadsworth et al. (15) observed no significant effect on growth of *Bacillus subtilis* and *Escherichia coli*. In addition, Van Voorhies et al. (16) depicted faster rates of larval growth and early egg laying in *C. elegans* in the BBR environment. It seems that the deep-underground environment can inhibit cell proliferation, enhance antioxidant capacity (5, 10–14), and somehow promote larval growth and early laying (16), or it may eventually have no obvious influence (15). To explain this inconsistency, the biological effects of deep-underground environments still need further study.

The DUGM involves not only cells, animals and plants, but also human beings themselves, which are naturally the core of this subject. Miners are the pioneers in the current deep-underground environment (17). At present, research mainly focuses on the occupational risk factors or subjective investigation of miners (17–22). In previous questionnaire surveys of miners in deep-underground wells, we noticed that 68–76% of miners complained about sleep disorders (23, 24), which was notably related to complicated underground environmental factors. The underground environmental traits include low background radiation, high temperature, high humidity, high atmospheric pressure, high carbon dioxide concentration, distinctive rock composition (high radon concentration) (1, 2, 25), and lower oxygen concentration (26), and may also involve environmental microorganisms (27, 28) or other unknown features. Other reports about sleep disorders in miners also confirmed our findings (29–31). However, there has been no previous study on the influence of sleep quality combined with a deep-underground environment on the changes in metabolites in miners. With this aim, we conducted metabolomics of urine obtained from deep miners of the Jiapigou Minerals Limited Corporation of China National Gold Group Corporation (CJEML) to explore potential biological effects and possible sleep-related metabolic diversities and mechanisms.

## Materials and methods

### Study subjects

Twenty-seven subjects worked in a maximum depth of 1,500 m from CJEML were included in this study during October 2019, whereas 12 local aboveground workers were recruited as control.

The volunteers in the deep-underground group (DU) required those who had been working underground for at least 1 month, and the volunteers in the aboveground group required that they had not been in the underground environment for nearly 1 month or never. The exclusion criteria were that the participating miners failed to complete the questionnaire and collect urine samples. Written informed consent was obtained from all participants, and all personally identifiable information was concealed.

### Questionnaire survey

The Pittsburgh Sleep Quality Index (PSQI) (32), was used to investigate the actual sleep status of the subjects in the past month. In this study, two investigators received standardized training in advance, and were responsible for the corresponding questionnaire survey. The questionnaire collected essential information about the characteristics of people in the above ground and deep-underground groups, including age, depth of the workplace, duration and other basic information related to working environment and sleep quality. After collecting all relevant information of volunteers, all questionnaire information was collated and summarized, the PSQI score was obtained according to specific calculation rules, and corresponding sleep grouping and subsequent analysis were performed. The PSQI was an important basis for the subsequent analysis and grouping of this study.

### Urine collection

The first morning urine samples of volunteers were collected in sterile containers of approximately 30 to 50 ml before their underground work. Then, the samples were temporarily stored in a 4°C low-temperature refrigerator, and centrifuged in a 4°C low-temperature centrifuge at 1,000 revolutions per minute (rpm) for 20 min, and then the supernatant was stored in a 15 ml centrifuge tube. Each sample was split into two centrifuge tubes. Subsequently, the samples were immediately stored in dry ice and stored in a −80°C ultralow temperature refrigerator. Urine was used to conduct liquid chromatography-mass spectrometry (LC-MS) non-targeted metabolomics analysis.

### UPLC-triple-TOF-MS analysis

The metabolomics analysis was performed by OE biotech Co., Ltd. (Shanghai, China). The analytical instrument used in this experiment was a LC-MS system composed of an AB ExionLC ultrahigh performance liquid chromatograph (UPLC, Waters Corporation, Milford, USA) in tandem with an AB Triple TOF 6,600 high resolution mass spectrometer (AB SCIEX Framingham, MA).

The combined system was used to analyze the metabolic profiling in both electrospray ionization (ESI) positive (ESI+) and ESI negative (ESI−) ion modes. An ACQUITY UPLC BEH C18 column (100 mm × 2.1 mm, 1.7 μm) was employed in both positive and negative modes. Data acquisition was performed in full scan mode (m/z ranged from 70 to 1,000) combined with information-dependent acquisition (IDA) mode. For IDA analysis, the range of m/z was set as 50–1,000, and the collision energy was 30 eV. The quality control samples (QCs) were injected at regular intervals (every 8 samples) throughout the analytical run to provide a set of data from which repeatability can be assessed.

### Data preprocessing

The LC-MS raw data obtained were analyzed by Progenesis QI software (Waters Corporation, Milford, USA) with the following parameters. Precursor tolerance was set at 5 parts per million (ppm), fragment tolerance was set at 10 ppm, and retention time (RT) tolerance was set at 0.02 min. Internal standard detection parameters were deselected for peak RT alignment, isotopic peaks were excluded for analysis, and noise elimination level was set at 10.00, and the minimum intensity was set to 15% of the base peak intensity. The Excel file was obtained with three dimensional data sets including m/z, peak RT and peak intensities, and RT-m/z pairs were used as the identifier for each ion. By removing any peak with a missing value (ion strength = 0) exceeding 50% in the sample, the resulting matrix was further reduced. The internal standard was used for data quality control (reproducibility).

Metabolites were identified by Progenesis QI (Waters Corporation, Milford, USA) Data Processing Software, based on public databases (such as https://www.hmdb.ca/, https://www.lipidmaps.org/) and self-built databases of OE biotech Co., Ltd. (Shanghai, China).

The raw data from OE biotech Co., Ltd. (Shanghai, China) were further processed under strict conditions. Ion peaks with missing values (ion strength = 0) exceeding 20% in the group were deleted, and then metabolites lower than the detected value or undetected were replaced with the lowest concentration. In addition, the identified compounds were screened according to qualitative score of the compounds. The screening standard was 40 points (out of 60 points). If the score was <40 points, the qualitative results were considered inaccurate and eliminated. Furthermore, the coefficient of variation of each metabolite or characteristic ion peak in QC samples was limited to <30%. Finally, the ESI+ and ESI− ion peak data were merged into a new matrix, which was the basis of all subsequent analyses.

### Statistical analysis

Raw data were further analyzed by R (v.4.0.5) and SIMCA 14.1. Principal component analysis (PCA) and orthogonal partial least-squares-discriminant analysis (OPLS-DA) were carried out to visualize the metabolic alterations among experimental groups after unit variance (UV) scaling. Hotelling's T2 range, shown as an ellipse in score plots of the models, defined the 95% confidence interval of the modeled variation. Variable importance in projection (VIP) ranks the overall contribution of each variable to the OPLS-DA model, and those variables with VIP larger than 1 are considered relevant for group discrimination. A permutation test (*n* = 200) was conducted to guard against overfitting. The quality of the model was evaluated by its explicative (*R*^2^) and predictive (*Q*^2^) abilities.

First, a test of normality was conducted to determine the distribution of the data. Then the Mann-Whitney U test was applied since the assumption of normality was questionable. The differential metabolites were selected on the basis of the combination of a statistically significant threshold of VIP values obtained from the OPLS- DA model and *p*-values from a Mann-Whitney U test, where metabolites with VIP values larger than 1.0 and *p*-values < 0.05 were considered potential differential metabolites. Since the potential “information-rich” molecular features were preserved in this study, the unadjusted *p*-value was finally used (33, 34). The final qualified differential metabolites also needed to meet the condition of fold change (FC) larger than 1.2 or FC <0.83.

### Potential biomarker identification

The identification of those ion peak characteristics selected by the OPLS-DA analysis was conducted by querying their exact mass against those presented in the online Human Metabolome Database (HMDB) v.4.0 (http://www.hmdb.ca/), Lipid Maps Database (https://www.lipidmaps.org) and the Metlin Database (https://metlin.scripps.edu). The following adducts were included: [M+H], [M+K], [M+Na], and [M+NH_4_] for ESI+ ionization mode, and [M-H] as well as [M+FA-H] for ESI− ionization mode. Neutral H_2_O loss was also taken into account for both ionization modes. Metabolite annotation was also supported by comparing the obtained LC-MS fragmentation spectra with those spectra experimentally proposed in these databases. Finally, the potential metabolic biomarkers were determined by combining the statistical significance of differential metabolites with the enrichment of the Kyoto Encyclopedia of Genes and Genomes (KEGG) pathway.

## Results

### Basic characteristics of the study subjects

Thirty-nine participants were recruited in this study, including 27 deep-underground workers as the experimental group and 12 ground volunteers as the control group. Table 1 illustrates the clinical characteristics of these study subjects. There was no significant difference in age, sex, PSQI score, alcohol intake, complaints of sleep disorder, self-evaluation of sleep quality, sleep time, other diseases, or night shift. In addition, different depths of the underground workplace were initially considered, but most of them (92.6%) were below 1,200 m. More importantly, the distribution of sleep quality was shown according to the PSQI score, and sleep quality was divided into two grades: good sleep quality (0–5), and poor sleep quality (>5).

**Table 1:** Basic characteristics of participants.

	**Ground**	**Deep-underground**	***p*-value**
	**(*N* = 12)**	**(*N* = 27)**	
Age, y^a^	41.17 ± 5.01	43.0 ± 7.73	0.458
Male sex, *N* (%)	12 (100%)	27 (100%)	-
Depth, *N* (%)			
0 m	12 (100%)	-	-
600–1,200 m	-	2 (7.4%)	-
>1,200 m	-	25 (92.6%)	-
PSQI^c^			0.957
0–5	5	13	-
6–10	4	7	-
11–15	2	4	-
>15	1	3	-
Smoker, *N* (%)	2 (16.7%)	13 (48.1%)	0.063
Drinker, *N* (%)	5 (41.7%)	18 (66.7%)	0.133
Complaints of sleep disorders	3 (25.0%)	7 (25.9%)	0.640
Self-evaluation of sleep quality			0.770
Very Good	3	6	-
Good	8	16	-
Poor	1	4	-
Very poor	-	1	-
Sleep time (h)^b^	6.88 ± 1.48	6.93 ± 1.99	0.937
Other disease, *N* (%)	2 (16.7%)	1 (3.7%)	0.219
With night duty, *N* (%)	2 (16.7%)	13 (48.1%)	0.063

^a, b^*The data were normally distributed, and the mean ± standard deviation (SD) was adopted*.

*^c^PSQI refers to the Pittsburgh Sleep Quality Index*.

### Metabolomics analysis

The original dataset (including ESI+ and ESI−) contains 6,181 measured variables. According to the strict data preprocessing method mentioned in the previous section, the following new matrices were obtained, including 1,389 metabolites. Different groups were compared to determine the influence of different environments and sleep quality on metabolites.

### Urine metabolic profile and differential metabolite expression

**Figure 1:**
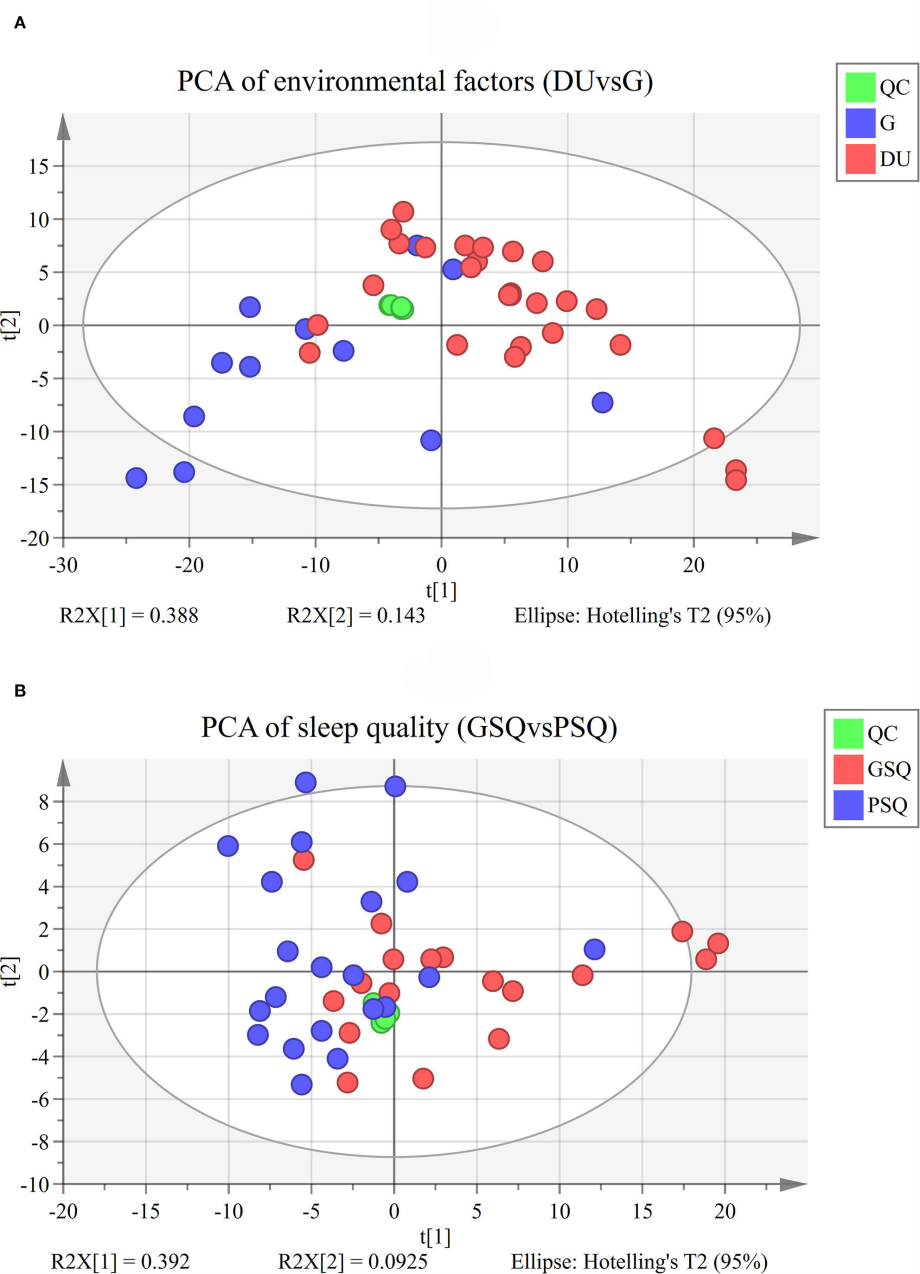
**(A)** PCA of the environmental factor group (DUvsG), DUvsG refers to the group “Deep-underground vs. ground,” and QC refers to the “quality control” samples. PCA refers to the principal component analysis; **(B)** PCA of the sleep quality group (GSQvsPSQ), GSQvsPSQ refers to the group “Good sleep quality vs. poor sleep quality,” and QC refers to the “quality control” samples. PCA refers to the principal component analysis.

**Figure 2:**
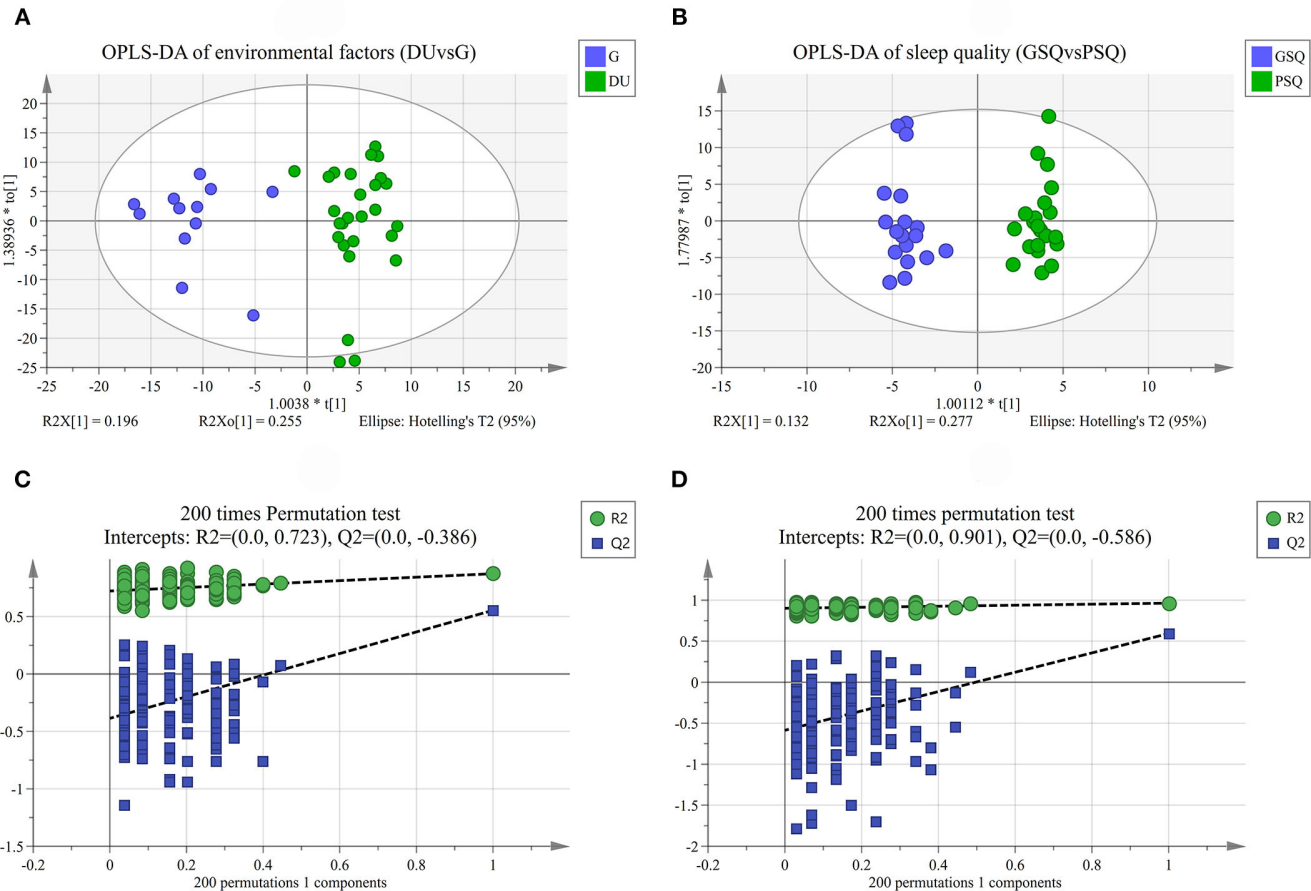
**(A)** is the OPLS-DA of environmental factors group (DUvsG), and DUvsG refers to the group “Deep-underground vs. ground”; **(B)** is the OPLS-DA of sleep quality group (GSQvsPSQ), and GSQvsPSQ refers to the group “Good sleep quality vs. poor sleep quality;” **(C)** is the results of 200 times permutation test in DUvsG group; **(D)** is the results of 200 times permutation test in GSQvsPSQ group. The quality of the model was evaluated by its explicative (*R*^2^) and predictive (*Q*^2^) abilities. OPLS-DA refers to the orthogonal partial least-squares-discriminant analysis.

Quality control samples assembled an exceedingly well-defined cluster as presented in the PCA score plot and Hotelling's T2. However, it should be noted that there were 4 outliers in the PCA of environmental factors, and 3 outliers in the PCA of sleep quality. Considering the potential information and sample size, we finally choose to retain the abnormal results. The results of PCA are shown in Figures 1A,B, and the results of OPLS-DA are shown in Figures 2A,B. OPLS-DA revealed a clear separation between deep-underground and ground and a clear separation between good sleep quality and poor sleep quality. At the same time, a 200 times permutation tests and the cross validation (CV) method were used to estimate the quality of the model. The fitting qualities of the two groups were both satisfactory. Parameters of OPLS-DA were observed, *R*^2^(Y) = 0.873, *Q*^2^(Y) = 0.554 and CV-ANOVA *p* < 0.001 in the deep-underground vs. ground (DUvsG) group, and parameters *R*^2^(Y) = 0.964, *Q*^2^(Y) = 0.593 and CV-ANOVA *p* < 0.01 in the good sleep quality vs. poor sleep quality (GSQvsPSQ) group. The detailed parameters of the permutation test, *R*^2^(Y)-intercept and *Q*^2^(Y)-intercept, are also shown in Figures 2C,D. *R*^2^(Y) measures the goodness of fit while *Q*^2^(Y) measures the predictive ability of the model (35). *Q*^2^(Y) > 0.5 is admitted for good predictability, and *Q*^2^(Y)-intercept < 0.05 is considered that the model is not overfitted.

**Figure 3:**
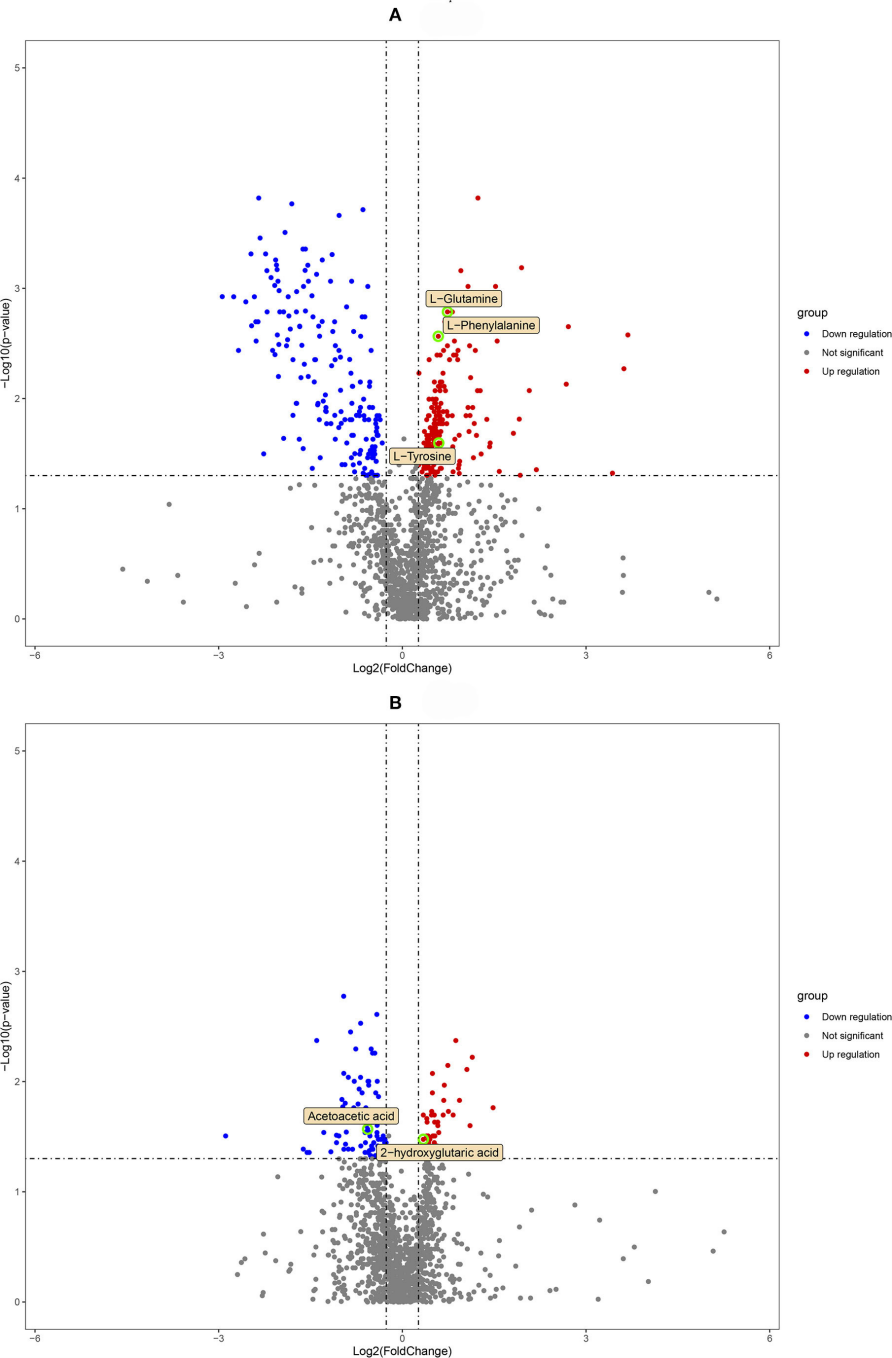
**(A)** is the volcano plot of the environmental factor group (DUvsG), and DUvsG refers to the group “Deep-underground vs. ground”; **(B)** is the volcano plot of the sleep quality group (GSQvsPSQ), and GSQvsPSQ refers to the group “Good sleep quality vs. poor sleep quality.” The points circled in green are the finally defined differential metabolites of potential biomarkers.

Qualified differential metabolites required VIP > 1.0, *p* < 0.05, and FC > 1.2 or FC <0.83. Visualization of the *p*-value and FC value was achieved by volcano plots, which was conducive to screening of differential metabolites. As shown in Figures 3A,B, we found 316 differential metabolites in the DUvsG group and 125 differential metabolites in the GSQvsPSQ group. The results of differential metabolites among groups are shown in Table 2. When all differential metabolites were combined, we found that 11 metabolites were repeatedly observed between DUvsG and GSQvsPSQ, but these metabolites did not appear in the KEGG enrichment pathway. It seems that there was no common pathway between the two groups.

**Table 2:** The results of differential metabolites in different groups.

**Group^a^ Expression**	**Up regulation** ^**b**^			**Down regulation**			**Total**
	**Total**	**ESI+**	**ESI−**	**Total**	**ESI+**	**ESI−**	
DUvsG	162	111	51	154	106	48	316
GSQvsPSQ	44	38	6	81	58	23	125

^a^*DUvsG refers to the group “Deep-underground vs. ground,” GSQvsPSQ refers to the group “Good sleep quality vs. poor sleep quality”*.

*^b^“ESI+” refers to “electrospray ionization positive” ion modes, and “ESI−” refers to “electrospray ionization negative” ion modes*.

**Figure 4:**
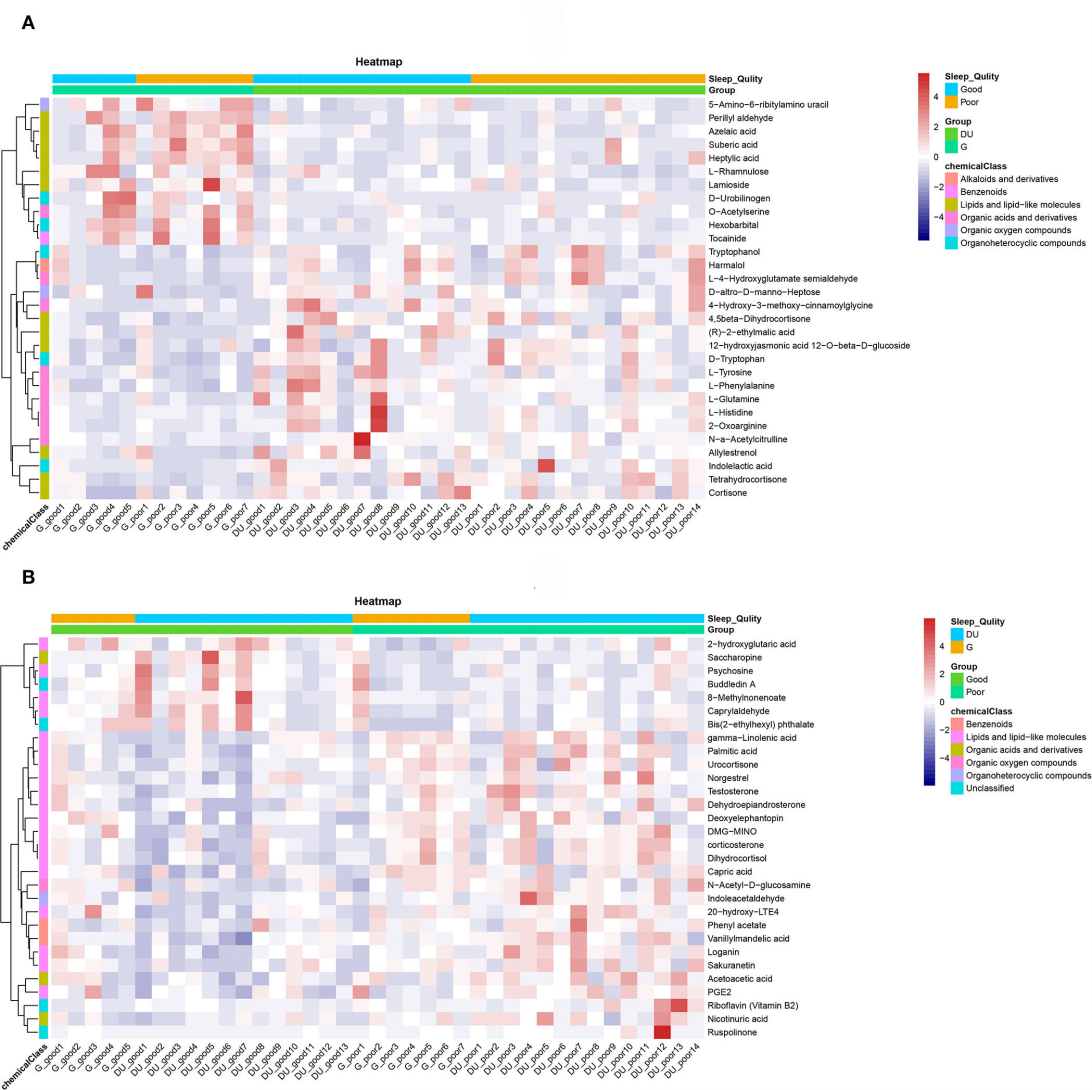
**(A)** Heatmap of the environmental factor group (DUvsG), and DUvsG refers to the group “Deep-underground vs. ground”; **(B)** Heatmap of the sleep quality group (GSQvsPSQ), and GSQvsPSQ refers to the group “Good sleep quality vs. poor sleep quality.” The abscissa represents the sample name, and the ordinate represents the differential metabolite. The color from blue to red signifies the expression abundance of metabolites from low to high, specifically, the redder indicats the higher expression abundance of differential metabolites. At the same time, information about different groups (deep-underground vs. ground, and good sleep quality vs. poor sleep quality) is labeled at the top.

**Figure 5:**
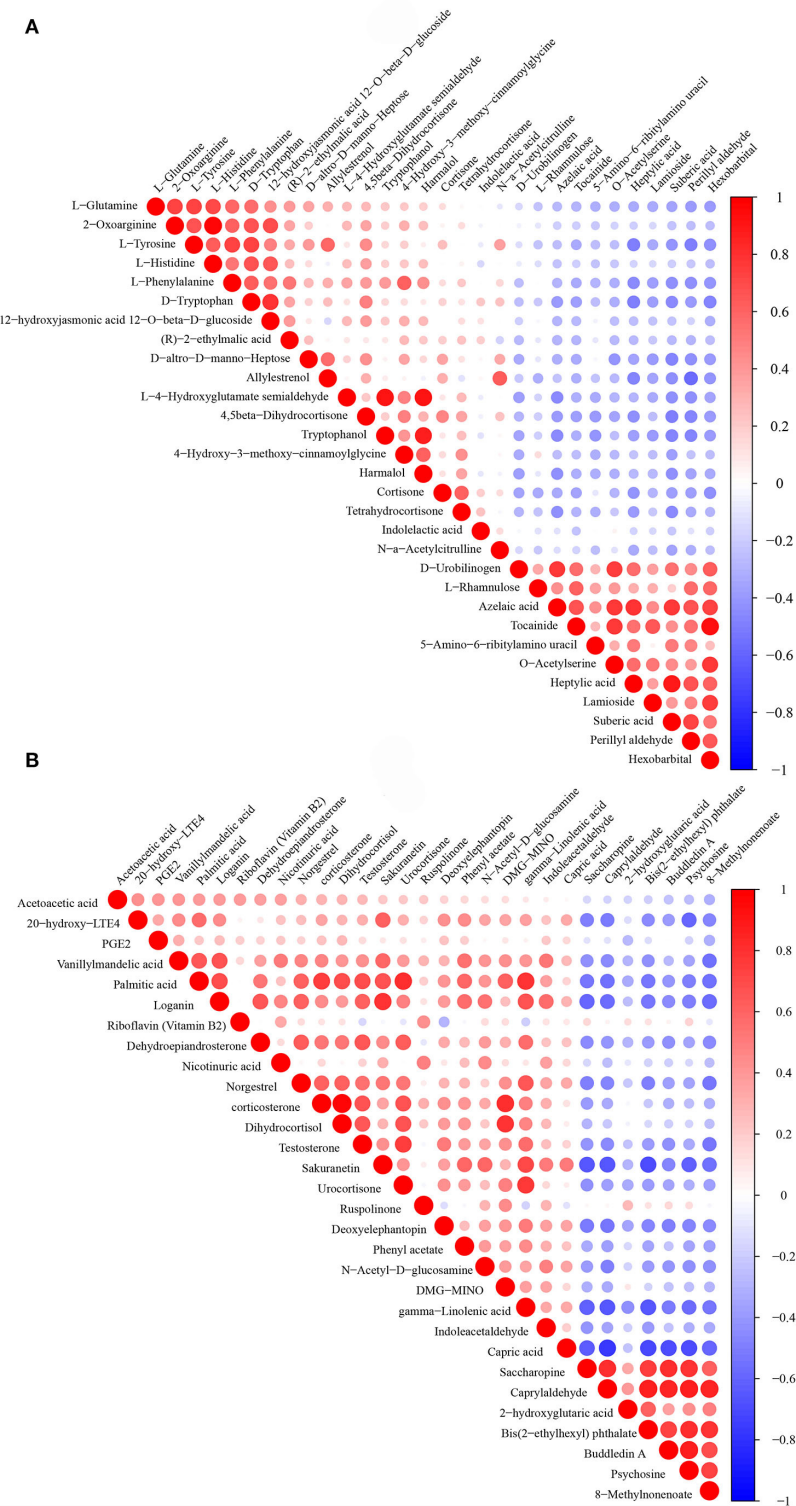
**(A)** is the Pearson correlation coefficient of the environmental factor group (DUvsG), and DUvsG refers to the group “Deep-underground vs. ground”; **(B)** is the Pearson correlation coefficient of the sleep quality group (GSQvsPSQ), and GSQvsPSQ refers to the group “Good sleep quality vs. poor sleep quality.” Red indicats a positive correlation, and blue indicats a negative correlation. The size of the dot represents the size of the correlation coefficient.

To show the relationship between samples and the differential metabolites as intuitively as possible, hierarchical clustering was adopted for all significant differential metabolites. According to the descending order of FC values, a total of 30 differential metabolites at both ends were analyzed. The results of the heatmap are shown in Figures 4A,B. The Pearson product-moment correlation coefficient, also known as the Pearson correlation coefficient, was used to measure the linear correlation between two quantitative variables. Correlation analysis directly showed the correlation and compactness of significant differential metabolites (only 30 metabolites are displayed in the figure). Figures 5A,B shows the relationship of differential metabolites in each paired group.

### Pathway analyses

**Figure 6:**
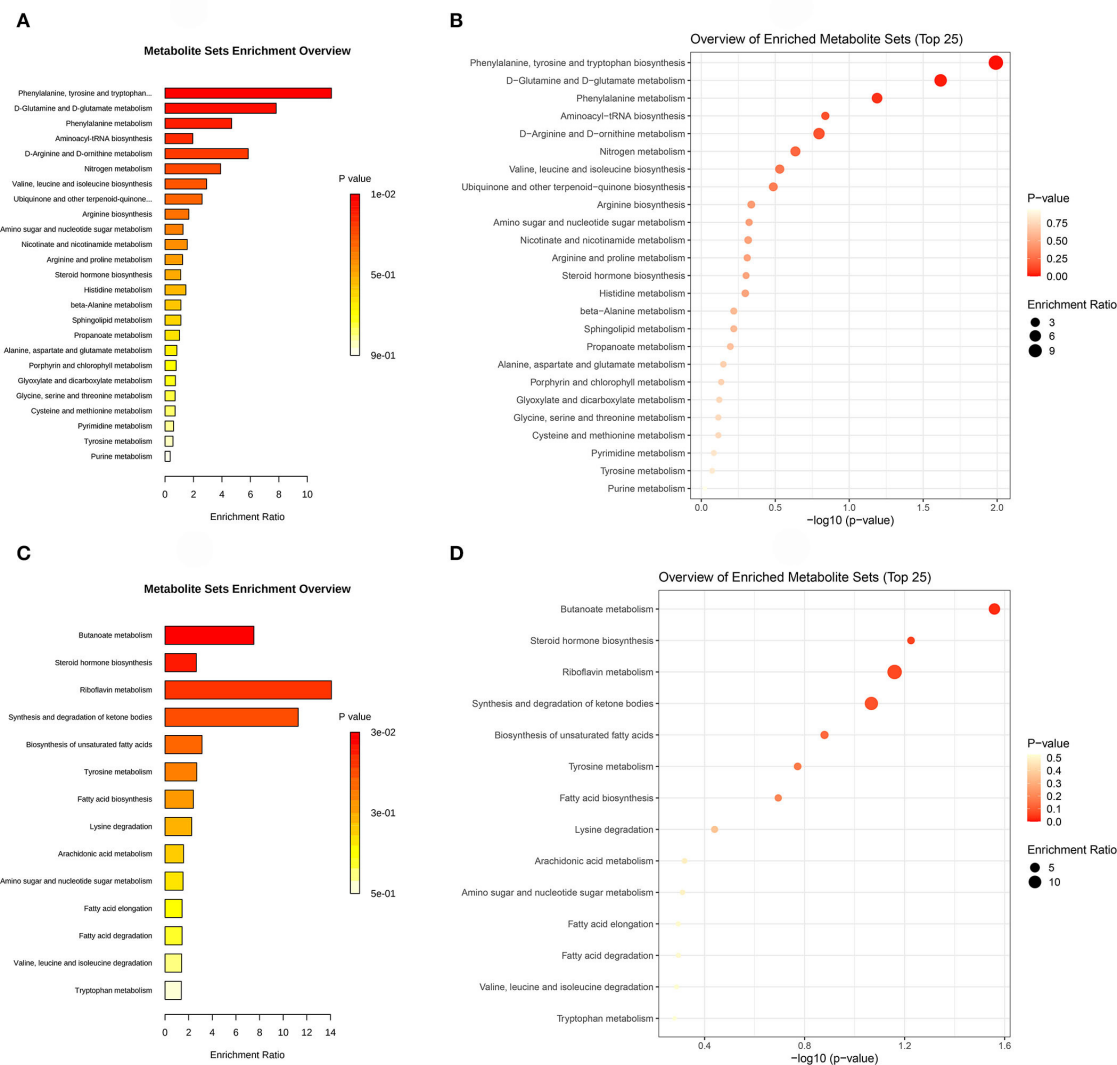
**(A)** is the KEGG pathway column chart of the environmental factor group (DUvsG), and DUvsG refers to the group “Deep-underground vs. ground”; **(B)** is the KEGG pathway bubble chart of DUvsG; **(C)** is the KEGG pathway column chart of the sleep quality group (GSQvsPSQ), and GSQvsPSQ refers to the group “Good sleep quality vs. poor sleep quality”; **(D)** is the KEGG pathway bubble chart of GSQvsPSQ. Each bar or dot represents a KEGG pathway. The left ordinate denotes the pathway names, and the abscissa indicates the enrichment ratio or the negative logarithm to the base 10 of *p*-value. Specifically, enrichment ratio indicated the ratio of the differential metabolites annotated to this pathway to the metabolites annotated to a certain pathway by this species. The higher the enrichment rate, the more reliable the enrichment significance of differential metabolites in this pathway.

Metabolic pathway enrichment analysis was based on the KEGG database, which was favorable for comprehending the metabolic pathway mechanism involved in differential metabolites. A hypergeometric test was used to determine the pathway items that were significantly enriched in the metabolites with significant expression differences compared with the whole background. Figure 6 shows the results of KEGG pathway analysis by column and bubble chart.

According to the ascending order of *p*-values calculated by hypergeometric analysis, the metabolic pathways of Phenylalanine, tyrosine and tryptophan biosynthesis (*p* = 0.0102) and D-Glutamine and D-glutamate metabolism (*p* = 0.0241) were significantly enriched between the deep-underground and ground groups (DUvsG). For GSQvsPSQ group, Butanoate metabolism was statistically significant (*p* = 0.0276). The details are listed in Table 3.

**Table 3:** Pathway enrichment results of the KEGG database.

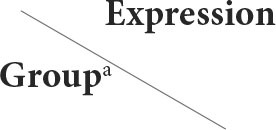	**KEGG**			
	**Pathway**	***p***	**KEGG compound**	**Metabolite name**
**DUvsG**	Phenylalanine, tyrosine and tryptophan biosynthesis	0.0102	C00079	L-Phenylalanine
			C00082	L-Tyrosine
	D-Glutamine and D-glutamate metabolism	0.0241	C00064	L-Glutamine
**GSQvsPSQ**	Butanoate metabolism	0.0276	C00164	Acetoacetic acid
			C02630	2-hydroxyglutaric acid

^a^*DUvsG refers to the group “Deep-underground vs. ground,” GSQvsPSQ refers to the group “Good sleep quality vs. poor sleep quality”*.

### Metabolite identification

Based on the metabolic pathways and metabolites involved, we identified the most discriminating metabolomics variables in DUvsG and GSQvsPSQ. Regarding the environmental factors, compared the deep-underground with the ground conditions, the origin of dissimilarity was most likely related to L-Phenylalanine, L-Tyrosine and L-Glutamine, and they were highly expressed in the underground group. With regard to sleep quality, the results indicated that sleep quality was most likely related to Acetoacetic acid and 2-hydroxyglutaric acid. Acetoacetic acid was highly expressed in the group with good sleep quality, while 2-hydroxyglutaric acid was highly expressed in the group with poor sleep quality. The identification of the most discriminant metabolomics variables in terms of environmental differences and sleep quality differences is illustrated in Supplementary Table 1.

**Figure 7:**
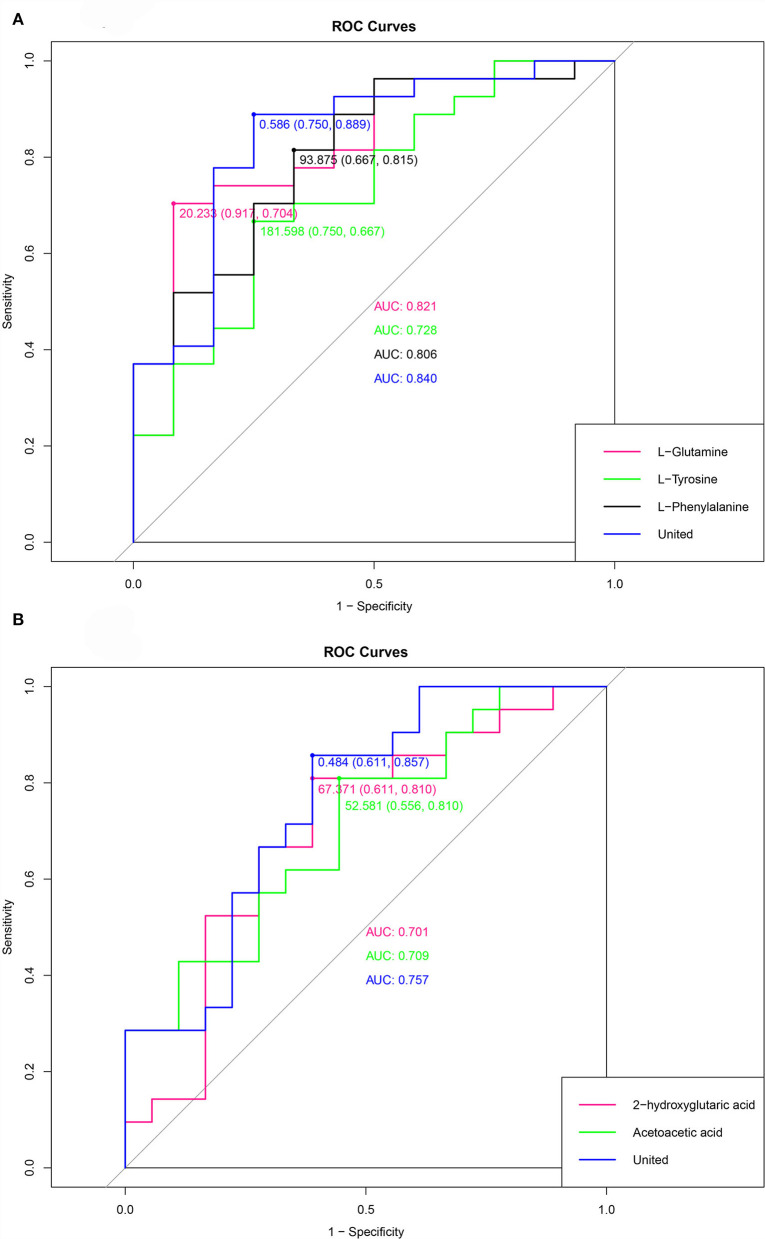
**(A)** is the ROC curve of the environmental factor group (DUvsG), and DUvsG refers to the group “Deep-underground vs. ground”; **(B)** is the ROC curve of the sleep quality group (GSQvsPSQ), and GSQvsPSQ refers to the group “Good sleep quality vs. poor sleep quality”. Different colors correspond to a metabolite, and “united” refers to the curve obtained by calculating several metabolites that were mentioned before.

The receiver operating characteristic (ROC) curve was a curve drawn with the true positive rate (sensitivity) as the ordinate and the false positive rate (1- specificity) as the abscissa. The ROC curves of environmental factors and sleep quality are depicted in Figure 7. The cutoff value, sensitivity, specificity and area under the curve (AUC) of metabolites in group DUvsG were as follows: L-Glutamine (20.233, 0.917, 0.704, and 0.821), L-Tyrosine (181.598, 0.750, 0.667, and 0.728), L-Phenylalanine (93.875, 0.667, 0.815, and 0.806), and the united ROC of the above three metabolites (0.586, 0.750, 0.889, and 0.840). The cutoff value, sensitivity, specificity and AUC of metabolites in group GSQvsPSQ were as follows: 2-hydroxyglutaric acid (67.371, 0.611, 0.810, and 0.701), Acetoacetic acid (52.581, 0.556, 0.810, and 0.709), and the united ROC of above two metabolites (0.484, 0.611, 0.857, and 0.757).

## Discussion

In recent years, the study of the biological impact of deep-underground laboratories has received increasing attention from international research teams (9, 25, 36–39). This study attempts to reveal the potential effects related to sleep quality and underground environmental factors through metabolomics research. Notably, we are the first group to report urine metabolomics of deep-underground miners. In this study, the involved miners routinely worked at depths of 645-1500 m, and 63% of them were below 1,400 m. We found that more metabolic variances were attributable to the differences between the underground and ground environments rather than the main effects of sleep quality. Admittedly, sleep quality is also affected by many factors, such as shift, aging, high temperature and hypoxia exposure (30, 40–44), but it is difficult to quantify for intricate underground workers. At least as important, smoking (45) and alcohol intake (46) have significant effects on sleep. In addition, both drinking and smoking habits are all related to caffeine intake, while higher caffeine intake is usually related to the poor subjective sleep quality (47). In this study, although there is no statistical difference between the underground and ground group, the numerical difference between smoking and drinking is obvious, which is worth considering. However, metabolomics analysis can also provide enough information to explore the characteristics and interactions behind it.

Through demographic classification, we divided 39 people into (1) the deep-underground group and the aboveground group; and (2) good sleep quality and poor sleep quality. Additionally, we conducted subgroup analysis according to ground and underground, and the results were basically consistent with the current analysis. In order to avoid the jumble of the displayed results, we did not make a separate description in this article.

Unsupervised PCA and supervised OPLS-DA are used for dimensionality reduction analysis to explore the differences between groups, and permutation tests and cross validation are used to evaluate whether the model is overfitting. The final results [*R*^2^(Y) = 0.873 and *Q*^2^(Y) = 0.554 in DUvsG; and *R*^2^(Y) = 0.964 and *Q*^2^(Y) = 0.593 in GSQvsPSQ] show preferable explicative and predictive abilities between groups. These results showed that the model has high compatibility and predictive power, which provides an effective guarantee for this research to study the influence of environmental factors and sleep quality. Under strict filtering criteria, there were 316 differential metabolites in the DUvsG group and 125 differential metabolites in the GSQvsPSQ group. As intuitively displayed in the volcano plots, the differences between the deep-underground group and ground group may be far > the variances between groups with different sleep quality. According to the results of heatmaps, the DUvsG group presents a better effect in distinguishing different groups, which is consistent with our previous inference.

When we analyzed the significant differential metabolites for KEGG pathway enrichment, we found three enriched pathways. In the group DUvsG, we observed that Phenylalanine, tyrosine and tryptophan biosynthesis and D-Glutamine and D-glutamate metabolism are prominent pathways, of which L-Phenylalanine, L-Tyrosine and L-Glutamine are considered key metabolites. Butanoate metabolism was the significantly enriched pathway in GSQvsPSQ group, of which Acetoacetic acid and 2-hydroxyglutaric acid were defined as chief metabolites. According to ROC, the AUC values of the 5 metabolites in the two comparisons were relatively high. It is worth mentioning that the sensitivity, specificity and AUC of L-Glutamine reached 0.917, 0.704, and 0.821, respectively, while the specificity of L-Phenylalanine was as high as 0.815. In contrast, the AUC values of potential sleep quality biomarkers were slightly lower but still higher than 0.7. Therefore, L-Glutamine and L-Phenylalanine can be potential and important biomarkers when distinguishing the ground and the deep-underground. Acetoacetic acid and 2-hydroxyglutaric acid are potential biomarkers to distinguish between good sleep quality and poor sleep quality, but they need to be considered.

Amino acid metabolism plays an important role in the central nervous system. Amino acids can regulate neuronal activity through a variety of mechanisms, ranging from the synthesis of neurotransmitters (such as precursor amino acids phenylalanine, tyrosine and tryptophan) to direct neurotransmission and neuromodulation (such as the excitatory neurotransmitter glutamate and the inhibitory neurotransmitter glycine) (48, 49). Phenylalanine, tyrosine and tryptophan biosynthesis signaling pathways are related to oxidative stress, immune response and inflammation regulation, and can also be used as energy compensation in extreme environments such as hypoxia (50). Phenylalanine can be converted into tyrosine, and tyrosine can further produce dopamine, norepinephrine and epinephrine. These excitatory neurotransmitters participate in the sleep-wake cycle and are important neurotransmitters that cause insomnia (51, 52). Furthermore, Glutamate is the most important and abundant excitatory neurotransmitter in the mammalian central nervous system (CNS) (53, 54). It promotes neurons to release chemical signals, which play a negative role in the regulation of sleep quality. In neurons, glutamate is synthesized from glutamine by glutaminase. After glutamate is released, it is taken up by astrocytes and converted into glutamine by glutamine synthetase. Subsequently, glutamine is transported and recycled in neurons. This glutamate–glutamine cycle is an indispensable component of the glutamatergic neurotransmission system (55). Allen et al. (56) mentioned that the level of glutamate/glutamine in the thalamus may be abnormally increased indicating an increase in glutamatergic activity, which may lead to awakening, night disturbance and sleep shortening. In this study, the concentrations of L-Phenylalanine, L-Tyrosine and L-Glutamine in deep-underground group were approximately 1.5 times those in the ground group. Combined with previous data (23, 24), the main complaints of miners are difficulty falling asleep and dreaminess, which is consistent with the influence of elevated metabolite levels. The high expression of excitatory neurotransmitters in the CNS of underground miners may be the core factor leading to sleep disturbance complaints.

Butanoate metabolism (ketone body metabolism) describes the metabolic fate of many short-chain fatty acids or short-chain alcohols, which are usually produced by intestinal fermentation (57, 58). Many of these molecules are ultimately used in the production of ketone bodies and short-chain lipids or as precursors of the citrate cycle, glycolysis or glutamate synthesis (57, 59). This means that Butanoate metabolism is closely related to energy metabolism. Ketone acetoacetate and β-hydroxybutyrate produced by fatty acid decomposition are the chief metabolic fuels for the brain under conditions of low glucose accessibility. Acetoacetic acid seems to play a fundamental role in the regulation of sleep homeostasis through the progress in central metabolism of ketones. A study carried out on mice showed that central acetoacetic acid injection significantly increased the slow-wave activity during NREM sleep, and stifled glutamate release (60). 2-hydroxyglutaric acid (2-hydroxyglutarate, 2HG) is also a part of the Butanoate metabolic pathway, and 2HG is a metabolite derived from α-ketoglutarate, which is related to changes in energy metabolism (61). 2-Hydroxyglutarate exists in 2 isomers: L-2HG and D-2HG. Their accumulation is associated with metabolic disorders termed 2-hydroxyglutaric aciduria and certain cancers (62–66). Williams et al. (61) found that L-2HG increased HIF-1α stability, thus increasing its activity. Subsequently, the increase in HIF-target gene expression contributes to the activation of proinflammatory factors, including the adoption of high glycolysis metabolism and the expression of HIF-1α -dependent genes, especially the gene encoding interleukin 1β (IL-1β). The low expression of Acetoacetic acid (FC = 0.6752) and the high level of 2HG (FC = 1.2683) in the underground environment seem to suggest that there is a change in high glycolytic ability. Meanwhile, the excitatory effect of neurotransmitters and the anti-inflammatory effect of the body may also be enhanced.

In this study, however, there was no significant difference in PSQI scores between the deep-underground group and the ground group except for the change in metabolic substances. The increased expression of L-Phenylalanine, L-Tyrosine and L-Glutamine in the deep-underground group was related to more complaints of sleep disorder, and the sample size of this study may be a vital reason for limiting the statistical difference. We speculate that under the stimulation of underground environmental factors, the metabolism of amino acids will be significantly enhanced, and the high arousal state of stimulation will be maintained by increasing the production of excitatory neurotransmitters, but this has not yet had a significant impact on the actual sleep quality. In addition, in both the DUvsG group and the GSQvsPSQ group, it seems that the pathways of significant enrichment and metabolites involved all indicate the regulation of excitatory neurotransmitters and the existence of abnormal energy metabolism related to hypoxia.

Most importantly, the underground environment is extremely complex, and it is difficult for us to attribute it to a single influence of a particular factor. Aforementioned environmental parameters showed below background radiation, higher temperature, higher humidity, higher atmospheric pressure, higher carbon dioxide concentration, and lower oxygen concentration in 1,470 m underground (3, 23). These parameters were all measured under the condition of ventilation (3, 24), and there may be some differences in actual experience. Besides, because of the challenging ventilation in a deep mine, radiation was the only environmental factor that could be kept at a constant level. As in this study, 14 underground miners (51.9%) complained about the high temperature, and 7 underground miners (25.9%) complained about poor ventilation and lack of oxygen. While our team's previous research showed that these proportions were about 33.5% and 32.4% (23), respectively. Therefore, these important issues should not be ignored when we consider the influence of underground environmental factors on the organism.

A limitation of this study is the small sample size, which leads to the unavoidable use of unadjusted *p*-values in the multiple comparisons analysis, but this has also been used in previous studies (33, 34). Notably, the periods of underground work as well as the living and dietary habits were not thoroughly taken into account in this study. Although we know that most of them work for several years and have long-term underground exposure, this has not been explicitly counted. In addition, miners work underground for 8 h each day, but nearly 50% of them work night shifts. As mentioned earlier, the impact of night shifts on sleep can't be ignored. Additionally, the stimulus of smoking and alcohol intake on sleep quality was also important, and these two influences can't be excluded. Admittedly, the depth of the deep-underground has not been clearly described, nor can we give a clear definition. However, we suppose that the definition of depth varies based on the development level of human science and technology. To some extent, we can consider that depths below 400–500 m seem to be the current shallow deep-underground space, and 500–1,500 m is the secondary deep-underground space. Furthermore, 1,500–4,800 m is deep-underground space, and ultradeep-underground space is almost difficult for human beings to reach directly during the present period. With increasing depth, the difference in environmental parameters will also be greater, and all current efforts are exploratory strategies to advance deeper into the earth. In addition, this study lacks further functional verification, which is limited by a deep-underground environment and economic factors, which leads to the inability to provide more credible evidence based on multiomics, cell or animal model experiments. This study is only a pilot study of deep-underground medicine research, and the results and conclusions can only provide potential references. However, the enriched pathways and differential metabolites suggested by this study will also be an important exploration direction for further study.

## Conclusions

This pilot study has also made some important discoveries. The influence of the deep-underground environment on the human body is more likely to induce specific amino acid metabolism processes, which is related to the high expression of excitatory neurotransmitters in the central nervous system, and all of these seem to have suggestive guiding effects on sleep-arousal regulation. According to the sleep quality grouping, we observed enhanced glycolysis metabolism, an increase in excitatory neurotransmitters and proinflammatory activation. The change in metabolites that affect sleep may precede physical symptoms. L-phenylalanine, L-tyrosine and L-glutamine, Acetoacetic acid and 2-hydroxyglutaric acid may be potential biomarkers of the deep environment and sleep quality, respectively.

## Data availability statement

The raw data supporting the conclusions of this article will be made available by the authors, without undue reservation.

## Ethics statement

The studies involving human participants were reviewed and approved by West China Hospital of Sichuan University Biomedical Research Ethics Committee. The patients/participants provided their written informed consent to participate in this study.

## Author contributions

QW and JZh contributed to the statistical analyses, data interpretation, and manuscript writing. XS, TM, and YL were involved in the development of the statistical framework and they reviewed the manuscript. YX, LW, JC, and JWe collected and prepared samples. JWu, JZo, SL, and JL were involved in the design of the study. SL and JL supervised the entire project and revised the final manuscript. All authors contributed to the article and approved the submitted version.

## Funding

This research was supported by grants from the 1.3.5 project for disciplines of excellence (Grant No. ZYJC21048) provided by West China Hospital, Sichuan University; the Open Fund of the Key Laboratory of Deep Earth Science and Engineering, Ministry of Education (Grant No. DESEYU 201902); the Research Fund of Health Commission of Sichuan Province (Grant No. 20PJ029 and Grant No. 21PJ022); Sichuan Soft Science Project (Grant No. 2022JDR0091); and the Fund of Sichuan Provincial Science and Technology Department (Grant No. 2021YJ0231).

## Conflict of interest

The authors declare that the research was conducted in the absence of any commercial or financial relationships that could be construed as a potential conflict of interest.

## Publisher's note

All claims expressed in this article are solely those of the authors and do not necessarily represent those of their affiliated organizations, or those of the publisher, the editors and the reviewers. Any product that may be evaluated in this article, or claim that may be made by its manufacturer, is not guaranteed or endorsed by the publisher.
